# Effects of Authoritarian Leadership on Employees' Safety Behavior: A Moderated Mediation Model

**DOI:** 10.3389/fpubh.2022.846842

**Published:** 2022-05-12

**Authors:** Dawei Wang, Li Wang, Shuangju Wei, Peng Yu, Haichao Sun, Ximing Jiang, Yixin Hu

**Affiliations:** ^1^School of Psychology, Shandong Normal University, Jinan, China; ^2^School of Education Science, Kashi University, Xinjiang Uyghur Autonomous Region, Kashgar Xinjiang, China; ^3^School of Teacher Education, Taishan College, Tai'an, China; ^4^Shengli Petroleum Engineering Yellow River Drilling Corporation, Dongying, China

**Keywords:** authoritarian leadership, safety behavior, trust in leadership, locus of control, moderated mediation model

## Abstract

Safety behavior is one of the focal concerns to occupational health researchers and safety managers. This study examined the relationship between authoritarian leadership and safety behavior based on social exchange theory and locus of control theory, and further explored the mediating role of trust in leadership and the moderating role of locus of control. In this study, a total of 636 employees from petroleum enterprises were recruited, with random sampling used to collect data in two stages. The result showed that: (1) Authoritarian leadership is significantly and negatively related to employees' safety compliance behavior and safety participation behavior. (2) Trust in leadership plays a partially mediating role in the impact of authoritarian leadership on employees' safety compliance behavior and safety participation behavior. (3) Locus of control moderates the first half of the pathway through which authoritarian leadership affects employees' safety behavior through trust in leadership. For externals, the negative effect of authoritarian leadership on their trust in leadership is stronger, which in turn decreases their safety compliance behavior and safety participation behavior.

## Introduction

World Health Organization statistics show that economic losses in terms of workplace-related fatalities, injuries, and illnesses account for 4–5% of the world's GDP. And 70% of this is caused by unsafe employee behavior (1). More unfortunately, millions of employees suffer workplace injuries and thousands lose their lives every year as a result (2, 3). The safety behavior of employees not only affects the socio-economic development and long-term operation of enterprises (4), but also has a bearing on people's lives and well-being and social harmony and stability. In China, both the Party Central Committee and government sectors attach great importance to work safety. For example, Chairman Xi Jinping has put forward the opinion in the report of the 18th National Congress of the Communist Party of China “ Strengthen the enterprise safety production infrastructure to curb serious accidents” (5). Therefore, it is necessary to conduct more research in the area of safety behavior.

In order to avoid losses due to the lack of safety behavior of employees, numerous researchers have explored the factors that influence safety behavior, among which organizational factor is a significant factor that influences safety behavior (6–10). Further, leadership, as an critical element of an organization, is also an essential factor in influencing employees' behavior and attitudes, and different types of leadership styles have been shown in most studies to be closely related to employees' safety behavior (11–15). However, the existing literature is biased toward the study of leadership styles such as transformational leadership and ethical leadership proposed based on the Western cultural context (16, 17), and less explores paternalistic leadership, which is based on traditional Chinese cultural and prevalent in Chinese companies (18). Zheng et al. (19) considered paternalistic leadership as a typical leadership style in the Chinese context and classified it into three dimensions: benevolent leadership, moral leadership, and authoritarian leadership. Among them, authoritarian leadership refers to the leader who focus on establishing their authority and demanding absolute obedience from employees, which is a leadership style that tends to be authoritarian and autocratic. Currently, there are few studies and mixed findings on authoritarian leadership and employees' safety behavior. Liu et al. (20) demonstrated that authoritarian leadership has a significant positive effect on highway drivers' safety behavior; a small number of other studies have shown that authoritarian leadership affected employees' safety behavior significantly and negatively (21–23). Therefore, exploring the influence of authoritarian leadership on employees' safety behavior in the Chinese context can not only enrich the research in the field of safety behavior, but also provide new ideas for corporate safety management practices.

In addition to examining the relationship between authoritarian leadership and safety behavior, this study also sought to explore the underlying mechanisms between them. Occupational health psychology researchers argue that occupational safety is embedded in psychosocial and interpersonal environments (24). Psychological explanations of the work environment can account for differences in employee health and safety that go beyond what can be explained by physical, biological, and chemical hazards (25). Authoritarian leadership, as a negative leadership style, with its authoritarian and high power distance, tends to make employees feel that the leader is impersonal and not easily communicated with (26–29), which may reduce employees' trust in the leader. The less employees trust their leaders, the less likely they will be conscientious and proactive in completing the work emphasized by their leaders and the organization, i.e., employees may slack off on the safe behavior valued by their leaders and the organization. Thus, employees' trust in leadership may mediate the relationship between authoritarian leadership and safety behavior. Furthermore, if authoritarian leaders act on safety behavior through employees' trust in leadership, is this effect influenced by individual factors? According to cognitive appraisal theory of emotion, an individual's cognitive appraisal of information determines his or her emotional response, and this process is influenced by intra-individual psychological factors (30). Locus of control, which is a kind of individuals' typical cognitive attributional preference, may influence employees' evaluations of authoritarian leaders and subsequently their trust in them. Therefore, we suggest that locus of control may moderate the relationship between authoritarian leadership and employees' safety behavior.

In summary, we examine whether authoritarian leadership negatively affects safety behavior and whether employees' trust in leadership mediates the relationship between them. In addition, we examine the moderating role of locus of control in this mediation model.

## Theory and Hypotheses

### The Relationship Between Authoritarian Leadership and Safety Behavior

Neal et al. (31) argued that safety behavior consists of two dimensions: safety compliance behavior and safety participation behavior. In this regard, safety compliance behavior refers to the ability of employees to work in accordance with established safety codes of conduct while engaged in safety-related tasks; safety participation behavior means that employees join in behaviors that contribute to safety management and safe operation out of their own initiative. Safety compliance behavior and safety participation behavior represent different goals of employees. Specifically, Safety compliance behavior is more akin to a passive compliance forced by the corporate system and is often seen as part of the employees' in-role behavior; while safety participation behavior means that employees take the initiative to participate in the construction of a safety climate and is always regarded as a kind of employees' extra-role behavior (32). Specifically in terms of the job content of petroleum enterprises, checking safety equipment routinely, wearing safety helmets and overalls during production, not bringing cell phones on the body during oil field site exploration, wearing alarm devices at all times when entering refineries, using copper tools (instead of iron tools) and other job behaviors are all safety compliance behavior, which are explicitly stipulated in the safety management regulations of petroleum enterprises and need to strictly enforced by oil employees; while behaviors such as proposing more feasible safety management methods based on one's work experience in weekly meetings, reminding colleagues with low safety awareness to operate production equipment safely in the workplace, using walkie-talkies to confirm colleagues' safety at all times, and calling on colleagues to actively participate in safety training are all safety participation behavior, which are not within the scope of safety management regulations but contribute to the construction of corporate safety management. And it is the spontaneous behavior of employees based on their own recognition of the importance of safety. In addition, specifically in terms of petroleum enterprise jobs, both front-line employees and management leaders are expected to perform safety compliance behavior and safety participation behavior, with front-line employees required to carry out actual safety work on a daily basis and management leaders being responsible for site safety supervision and the overall safety building of the company. petroleum enterprises have jobs such as refining position, extraction position, and substation operations position, all of which are expected to perform safety compliance behavior and safety participation behavior. Current research on the antecedent variables of safety behavior has mainly focused on leadership style (12, 14–16, 33). However, most of the existing research has been concentrated on transformational leadership, transactional leadership, ethical leadership, and empowering leadership. For example, it has been found that transformational leadership is positively related to employees' safety behavior and that this process is mediated by job stress, job strain (16, 34, 35); transactional leadership negatively affects employees' safety behavior by reducing organizational safety climate and employees' psychological empowerment (36); ethical leadership has a positive effect on employees' safety behavior (37); and empowering leadership is positively correlated with employees' safety behavior (38). Faced with authoritarian leadership, a leadership style that is unique and more prevalent in Chinese organizations and reveals the deep-rooted qualities of traditional Chinese culture (39–41), few scholars have investigated its relationship with safety behavior. Moreover, its relationship with two dimensions of safety behavior is yet to be explored.

Authoritarian leadership is the leadership type that best reflects the leadership style of Chinese society among the three dimensions of paternalistic leadership (39, 40, 42, 43). It fully expresses the idea of “the three cardinal guides and the five constant virtues” and “upper respect and lower inferiority” that have been emphasized in traditional Chinese Confucianism and feudalism for thousands of years (44). Viaing studying authoritarian leadership, Fan and Zheng (45) concluded that it should include four dimensions, namely, “authoritarian style, image modification, degradation of subordinates' abilities, and teaching behavior.” The “authoritarian style” means that the leader concentrates power in his own hands, closely monitors his employees and does not have emotional communicate with them; “image modification” means that leaders maintains a confident and authoritarian image of themselves in front of their employees; “degradation of subordinates' abilities” means that the leader devalues employees' abilities, does not accept their suggestions, and never praises them for their contributions; “teaching behavior” means that the leader sets high performance standards for employees and directly reprimands those who do not perform well.

All four dimensions of authoritarian leadership reflect strict hierarchical relationships, high power distance, and low emotional communication between leaders and employees, which would have an impact on employees' subjective perceptions psychological feelings and subsequently on their work behaviors, including safety behavior (46, 47). And we can explain it in terms of social exchange theory. Homans (48) argued that in the process of social interaction, people analyze the “cost” they pay and the “reward” they receive in a relationship, and satisfactory social relationships should follow the reciprocity rule, which ensures a balance between payoffs and rewards. The “cost” and “reward” here include not only tangible material things (e.g., money) but also intangible feelings (e.g., respect, understanding, love). The social determinants of health in the field of social medicine emphasize the important influence of psychological and social interpersonal factors on a person's overall health. The behaviors of authoritarian leaders are not conducive to the performance of employees' safety behavior, which in turn can threaten the overall health of employees. Specifically, in authoritarian leadership, the leader strictly dominates and controls employees, rarely interacts with them in a positive way, and does not empower them; besides, the leader often belittles employees' abilities and reprimands them severely when their performance is not satisfactory, lacking encouragement and guidance. Consequently, these can surely cause negative emotions and a series of negative cognitions of employees (49–51). Employees' perceived organizational support (22) and psychological empowerment (52, 53) will decrease, and their organizational commitment, job satisfaction will also decline (54). As a result, employees may believe that they receive very limited “rewards” from their leaders and then choose to give less in return by reducing positive work behaviors or even displaying negative work behaviors. That is, employees will develop a sense of job alienation (28, 55, 56) and lower their work engagement (57, 58), which negatively affects their cognition and concentration of activities in terms of compliance with safety behavioral norms, etc.; in addition, employees will also increase their job insecurity (47), develop a rebellious mentality (59) and counterproductive work behavior (60), exhibiting behaviors such as slowdown, not following prescribed processes, and disregarding safety compliance regulations. Accordingly, we infer that authoritarian leadership has a negative effect on employees' safety compliance behavior. Similarly, these traits of authoritarian leadership will also reduce employees' innovativeness and initiative (47, 61, 62), decrease their organizational citizenship behavior (60, 63) and voice behavior (53, 64, 65). While safety participation behavior is the active behavior of employees who actively contribute to the organization's safety production, such as actively helping colleagues to operate production machines safely, advising in safety management activities, and consciously maintaining the company's safety environment. Obviously, employees under authoritarian leadership may be less likely to be willing to perform the safety participation behavior. Accordingly, we infer that authoritarian leadership also has a negative effect on employees' safety participation behavior. Therefore, the following hypotheses were proposed in this study.

Hypothesis 1a: Authoritarian leadership is negatively correlated with employees' safety compliance behavior.
Hypothesis 1b: Authoritarian leadership is negatively correlated with employees' safety participation behavior.

### The Mediating Role of Trust in Leadership

Trust is the basis for people's positive interactions and relationship-building (66). Trust in work situations plays an important role in facilitating coordination within organizations and achieving organizational strategic goals (67–69). And it has been received increasing attention from academic researchers and business managers. Rousseau (70) argued that trust is a psychological state that is based on positive expectations about the intentions and behaviors of others, and that people who trust others are willing to maintain a relationship with them and to take the risks that come with that relationship. McAllister (71) further classified trust into two dimensions: cognitive trust and affective trust. The former reflects an individual's trust in the reliability, dependability, integrity, and competence of others, and depends on a reasonable and objective assessment of the attributes of others (72); while the latter reflects the emotional connection between individuals and others, which is derived from the mutual care and concern between individuals and others (73). In the field of organizational behavior, employees' trust in leadership refers to the psychological state in which employees' willingness to the leaders in the work process is positive, and employees are willing to take risks for the leader and even accept the shortcomings of the leaders (70).

Currently, there are two different theoretical perspectives on the process of forming employees' trust in leadership: one is based on leader traits; the other is based on the leader-employee relationship. From the leader traits-based perspective, employees' perceptions of leaders' traits have a significant impact on the formation of trusting relationships between them. Employees tend to develop high levels of cognitive trust with leaders who are fair, reliable, sincere, kind, and competent (74). However, the autocratic, harsh, high power distance and impersonal qualities of authoritarian leadership are the opposite of the above qualities. When employees are confronted with authoritarian leaders, they may perceive them as unreliable and thus reduce their cognitive trust in them. In addition, the leader-employee relationship-based perspective focuses on the quality of the relationship between employees and leaders, because the quality of the relationship between superiors and subordinates depends on the level of trust between them (75, 76). When leaders show support, empowerment, care and concern for their employees, employees will increase their affective trust in their leaders (77, 78), which in turn facilitates the formation of good social exchange relationships between them. In authoritarian leadership, however, leaders are reluctant to empower their employees, rarely consider their needs and welfare, and refuse to communicate with them emotionally, which makes it difficult to develop higher quality exchange relationships between employees and leaders as they only remain in superficial economic exchanges (e.g., labor transactions). Naturally, the affective trust between them is also at a low level (27, 79, 80).

Trust is the basis of social exchange (81). Low level of trust between authoritarian leaders and employees is detrimental to the formation of high-quality exchange relationships between them (82, 83), and subsequently triggers a series of negative work attitudes and behaviors among employees. Studies have shown that employees' low level of trust in their leaders decreases their job satisfaction and work engagement (74, 84). Besides, employees' degree of effort, conscientiousness, and efficiency in completing tasks assigned by their leaders are also reduced (85), and employees are less able to produce the results expected by their leaders (86). Accordingly, we infer that when employees have low level of trust in their leaders, they are more likely to fail to comply with their leaders' safety requirements as well as the company's safety regulation and engage in less safety compliance behavior. In addition, Choi (87) showed that the level of trust between employees and leaders positively affects employees' psychological security; Burke (88) found that high level of employees' trust in leaders increases their willingness to provide more quality services to the organization; Pappas et al. (89) argued that employees' trust in leadership enhances their intention to take risks and promotes their greater involvement in the organization's strategic decision-making process. This means that low level of trust in leaders also decreases employees' psychological security and willingness to better serve the organization. And employees may be less likely to voluntarily and proactively engage in behavior that facilitates safety management and operations, such as actively contributing ideas about safety management in the workplace and urging colleagues to operate production equipment safely. From this we infer that low level of trust between employees and leaders also negatively affects employees' safety participation behavior. Therefore, the following hypotheses were proposed in this study.

Hypothesis 2a: Employees' trust in leadership will mediate the relationship between authoritarian leadership and employees' safety compliance behavior.
Hypothesis 2b: Employees' trust in leadership will mediate the relationship between authoritarian leadership and employees' safety participation behavior.

### The Moderating Role of Locus of Control

Cognitive appraisal theory of emotion suggests that the mechanism underlying emotional responses can be viewed as a cognitive appraisal mechanism of emotions, i.e., individuals' cognitive appraisal of information determines their emotional response, and that this appraisal process is influenced by intra-individual psychological factors (differences in beliefs, attitudes, personalities) (30, 90, 91). This results in large differences in the emotional experiences that people produce when faced with the same stimulus information (92, 93). That is, employees will have different emotional experiences of authoritarian leaders because their evaluations of authoritarian leaders vary. For example, it has been shown that authoritarian leadership can be evaluated as either “authoritarian” motivation, which emphasizes the control and discipline harshly, or “strict” motivation, which emphasizes the respect and focus on systems and norms (94–96), in which employees' individual differences play an important role.

Rotter's locus of control theory is a good explanation for the individual differences in employees' evaluations of authoritarian leaders (97, 98). Locus of control is the degree to which individuals perceive the outcome of behavior as internal or external. It is classified into two categories: internals (i.e., employees with internal or high LOC) and externals (i.e., employees with external or low LOC). Internals tend to believe that the outcome of behavior depends on their own actions (99), whereas externals tend to believe that the outcome of behavior depends on external factors beyond their control, such as the power of others, luck, or the environment (100–102). Research has shown that when faced with authoritarian leaders, who are characterized by “demeaning subordinates' abilities and relentless teaching and reprimanding,” internals tend to believe that they are responsible for the abusive behavior of authoritarian leaders (103), because they may attribute it to their poor performance or failure to satisfy the leader in other aspects of their work. In addition, internals usually believe that they have the ability to influence future outcomes (100, 104). Therefore, under the indignity of authoritarian leaders, they will experience more of a sense of failure that they failed to prevent or control the occurrence of abusive behavior by authoritarian leaders and a sense of shame that the image of the dedicated employee has been tarnished (105). However, internals also have a high sense of control and self-efficacy (106–110). They will behave proactively and dominate things to happen in their controllable direction when they perceive negative events. For example, employees will work harder to improve their performance and increase communication with their leaders (105, 111, 112) to repair their relationship with their leaders and reshape the image of dedicated employee, etc., all of which will contribute to enhancing their trust with authoritarian leaders. Therefore, we believe that internals may be more likely to build trust with authoritarian leaders and more willing to trust them than externals.

In contrast, externals tend to perceive themselves as having little responsibility for the abusive behavior of authoritarian leaders (104). And they often have a stronger self-serving bias, i.e., they are inclined to consider themselves victims when things go wrong and blame external factors, e.g., the abusive behavior of authoritarian leaders may be caused by the leader's personal emotions or personality (111, 113). Thus, the victim mentality of the externals, which is motivated by self-protection, saves them from feelings of self-blame and shame for the negative events (99). What's more, the externals also believe that they cannot cope with the current situation and cannot control the future development of the events (111). Also faced with low quality relationships with authoritarian leaders, externals usually suffer from greater psychological stress (114) and have lower self-efficacy (106) than internals. They will have less confidence to act proactively to try to achieve positive results, and choose to be negative or just avoid it. Externals' cognitive blame and negative behavior toward authoritarian leaders can weaken their trust with authoritarian leaders. From this we argue that externals will be less prone to build trust with authoritarian leaders than internals. Therefore, the following hypotheses were proposed in this study.

Hypothesis 3: Locus of control will moderate the first half of the pathway by which authoritarian leadership affects employees' safety behavior through employees' trust in leadership. For externals, the negative effect of authoritarian leadership on their trust in leadership will be stronger, which in turn will decrease their safety compliance and safety participation behavior.

**Figure 1:**
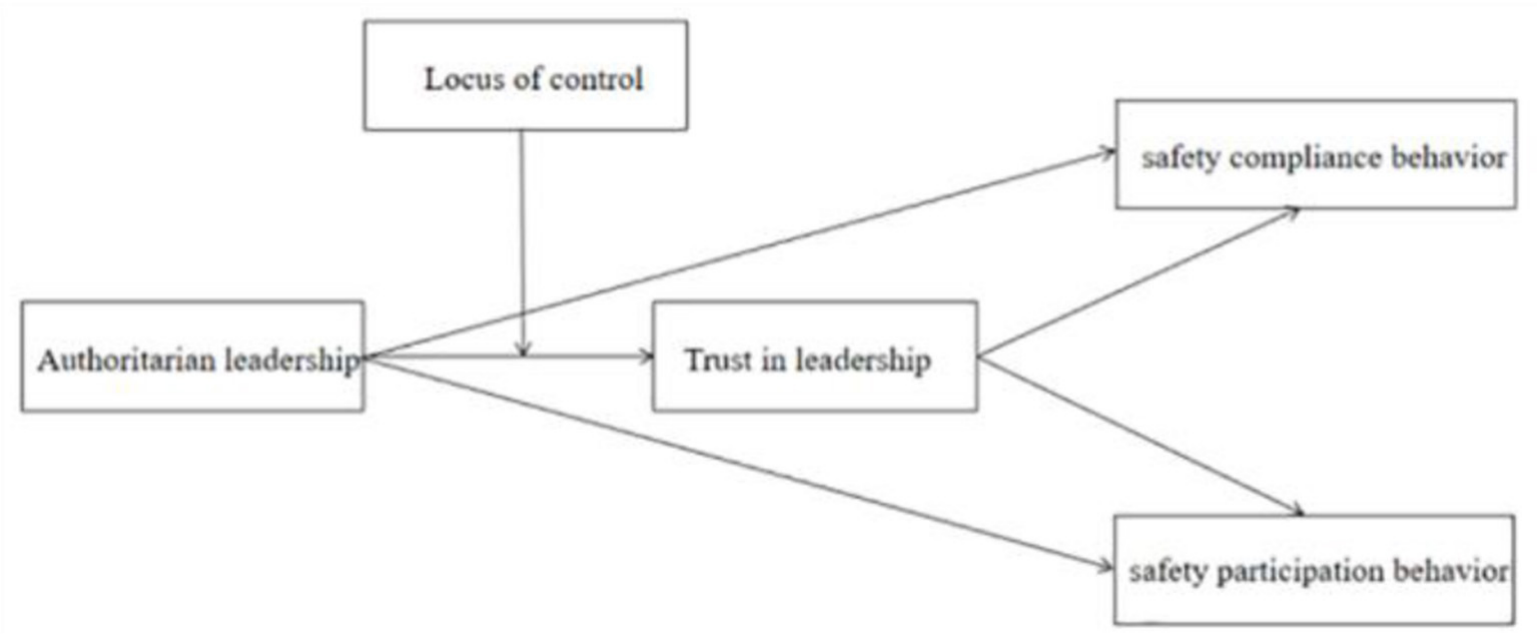
The hypothesized model.

In summary, based on social exchange theory and locus of control theory, this paper explored the relationship between authoritarian leadership and employees' safety compliance behavior and safety participation behavior. In addition, this paper also examined the mediating role of employees' trust in leadership and the moderating role of locus of control in order to examine the intrinsic mechanism of authoritarian leadership on employees' safety behavior, as shown in Figure 1.

## Methods

### Sample and Procedures

The study was conducted in petroleum enterprises. In order to improve sample representativeness and facilitate sample selection, we used a random sampling method to select oil workers in different oil work bases, and the administration sites and administration time were arranged by the head of each site. With different oil working bases as units, the person in charge of each unit unified to arrange the measurement staff, measurement location and measurement time. A total of 700 questionnaires were distributed, and 636 valid questionnaires were returned, with a recovery rate of 90.86%. Of the total sample respondents, 41.2% were male and 58.8% were female. With regard to age, the minimum age of the sample respondents was 23 years old and the maximum age was 60 years old, with a mean age of 40.27 years (SD = 6.30), 5.8% were under 30 years old, 18.3% were 31–35 years old, 29.7% were 36–40 years old, 23.6% were 41–45 years old, 18.2% were 46–50 years old, and 4.4% were over 51 years old. Regarding years of working, the shortest working years of sample respondents is 1 year, the longest working years is 43 years, the average working years is 18.91 years (SD = 8.34), 22.5% had worked for <10 year, 32.8% for 11–20 years, 37.6% for 21–30 years, 6.9% for 31–40 years, and 0.2% for more than 41 years. In terms of education level, 0.9% had a junior high school degree or less, 31.6% had a high school or junior college degree, 30.3% had a college degree, 27.4% had a bachelor's degree, and 1.7% had a master's degree or higher. Besides, 95% sample respondents were married and the distribution of positions was dominated by front-line employees (77.7%).

To avoid common method bias, a two-stage data collection approach was adopted. The first phase collected authoritarian leadership and employee trust in leadership, and the second phase collected psychological sources of control and safety behaviors, with a 1-month interval before and after. The study received ethical approval from the academic committee of the authors' university and complied with the 1964 Declaration of Helsinki. The surveys were conducted with the consent of the company's human resources department and the employees themselves, and informed consent forms were signed.

### Measures

#### Safety Behavior Scale

The safety behavior scale, developed by Neal et al. (14) and revised by Ye et al. (115), measures two dimensions including “safety compliance behavior” and “safety participation behavior.” Sample items include “I strictly abide by the safety rules and regulations in my work,” “I actively make suggestions that are conducive to working safely.” The 11-item questionnaire is rated on a 7-point scale ranging from 1 (totally disagree) to 7 (totally agree), with higher scales indicating a higher level of safety behavior. In this study, the Cronbach's alpha coefficient of the safety behavior scale was 0.96, among which the Cronbach's alpha coefficient of the safety compliance behavior subscale was 0.96 and the Cronbach's alpha coefficient of the safety participation behavior subscale was 0.955.

#### Authoritarian Leadership Scale

Authoritarian leadership was assessed using a 5-item questionnaire developed by Zheng et al. (19, 116), and revised by Fu et al. (117). One simple item was “My leader decides everything in the company alone.” Employees rated items on a 5-point liket scale ranging from 1 (totally disagree) to 5 (totally agree), with higher scores indicating a higher degree of authoritarian leadership. In this study, the Cronbach's alpha coefficient for the authoritarian leadership scale was 0.822.

#### Trust in Leadership Scale

The trust in leadership scale was developed by Nyhann and Marlow (118) and revised by He (119). One simple item was “My supervisor has a good understanding and grasp of his job tasks.” The 6-item questionnaire is rated on a 6-point scale ranging from 1 (totally disagree) to 6 (totally agree), with higher scales indicating employees' higher level of trust in leadership. In this study, the Cronbach's alpha coefficient for the trust in leadership scale was 0.894.

#### Locus of Control Scale

The locus of control scale was developed by Spector (120) and revised by Jiang et al. (121). The scale has 16 items, 8 of which are internals type, with simple item such as “Generally speaking, people who work hard get paid what they deserve,” eight of which are externals type, such as “Making money mainly depends on fortune.” Employees rated items on a 6-point liket scale ranging from 1 (totally disagree) to 6 (totally agree), with higher scales indicating employees' higher locus of control after reverse scoring. In this study, the Cronbach's alpha coefficient of the locus of control scale was 0.824.

#### Control Variables

Based on previous related research, this paper used employees' gender, age, education, and years of working as control variables to examine the extent of their influence on employees' safety behavior (122, 123).

## Results

### Common Method Bias

In this study, authoritarian leadership, trust in leadership, safety behavior, and locus of control scales were used as employee self-reported methods, and therefore common method bias may exist. Therefore, we conducted another test for common method bias by Harmam one-way test method based on the two-stage data collection. The results of exploratory factor analyses (EFAs) showed that a total of seven factors with characteristic roots >1 were extracted, and the maximum factor variance explained was 27.37% (<40%). Therefore, there was no serious common method bias in this study (77, 123).

### Confirmatory Factor Analyses

We used Mplus 8.0 to test the discriminant validity of the variables by performing confirmatory factor analyses (CFAs) on the observed data. The measurement model fitted the data acceptably (χ^2^/df = 1.559, RMSEA = 0.042, SRMR = 0.040, CFI = 0.973, TFI = 0.968). We further investigated several substitute measurement models and compared them with the five-factor model. As shown in Table 1, the five-factor model fits our data better than other models, suggesting that our respondents are able to distinguish the main constructs clearly.

**Table 1:** Results of confirmatory factor analysis of the measurement models.

**Measurement models**	***χ^2^***	***df***	***χ^2^/df***	**RMSEA**	**CFI**	**TFI**	**SRMR**
Five-factor model (A, B, C, D, E)	246.312	158	1.559	0.042	0.973	0.968	0.040
Four-factor model (A,B,C,D+E)	1096.616	224	4.896	0.111	0.774	0.744	0.100
Three-factor model (A,B,C+D+E)	1999.123	227	8.807	0.157	0.540	0.488	0.147
Two-factor model (A,B+C+D+E)	2007.320	229	8.766	0.156	0.539	0.490	0.147
Single factor model (A+B+C+D+E)	2458.498	230	10.689	0.175	0.422	0.364	0.164

*A, authoritarian leadership; B, locus of control; C, trust in leadership; D, safety compliance behavior; E, safety participation behavior*.

### Descriptive Statistics and Correlation Analysis

The results of the descriptive and correlational analyses showed (see Table 2) that authoritarian leadership is significantly and negatively related to safety compliance behavior and safety participation behavior (*r* = –0.186, *p* < 0.01; *r* = –0.158, *p* < 0.01). And it is also significantly and negatively related to trust in leadership (*r* = –0.138, *p* < 0.01). Besides, there is a significant and positive correlation between trust in leadership and safety compliance behavior and safety participation behavior (*r* = 0.354, *p* < 0.01; *r* = 0.306, *p* < 0.01). This analysis thus supports hypothesis 1a and hypothesis 1b.

**Table 2:** Descriptive statistics and correlations among study variables.

**Variable**	**M ±SD**	**1**	**2**	**3**	**4**	**5**	**6**	**7**	**8**	**9**
1. Gender	1.588 ± 0.493	–								
2. Age	40.267 ± 6.302	0.009	–							
3. Education	2.893 ± 0.876	−0.069	−0.467^**^	–						
4. Working years	18.892 ± 8.345	0.071	0.883^**^	−0.541^**^	–					
5. Authoritarian leadership	3.124 ± 0.795	−0.071	0.039	−0.015	0.014	–				
6. Trust in leadership	4.744 ± 0.769	0.104^**^	0.064	−0.029	0.069	−0.138^**^	–			
7. Locus of control	3.754 ± 0.656	0.028	−0.053	0.061	−0.080^*^	0.327^**^	−0.107^**^	–		
8. Safety compliance behavior	6.482 ± 0.824	0.094^*^	0.127^**^	−0.087^*^	0.188^**^	−0.186^**^	0.354^**^	−0.091^**^	–	
9. Safety participation behavior	6.229 ± 0.914	0.043	0.057	−0.088^*^	0.130^**^	−0.158^**^	0.306^**^	−0.037	0.772^**^	–

*N, 636; M, mean; SD, standard deviation; gender coded as (1 = male, 2 = female); education coded as (1 = junior high and below, 2 = high school or technical school, 3 = junior college, 4 = undergraduate, 5 = master or above);*

* *p < 0.05;*

** *p < 0.01*.

### The Mediating Effect of Trust in Leadership

According to Hayes (124, 125), we used the PROCESS program of SPSS 21.0 and selected Model 4 to test the mediating effect, with authoritarian leadership as the independent variable, safety compliance behavior and safety participation behavior as the dependent variables, and trust in leadership as the mediating variable, while incorporating gender, age, education, and working years as control variables. As shown in Table 3, authoritarian leadership has a significant and negative influence on safety compliance behavior (β = –0.149, *p* < 0.001), safety participation behavior (β = –0.139, *p* < 0.001); and it also has a significant and negative influence on trust in leadership (β = –0.126, *p* < 0.001); trust in leadership significantly and positively affects safety compliance behavior (β = 0.345, *p* < 0.001), and safety participation behavior (β = 0.341, *p* < 0.001). That is, trust in leaders partially mediated the effect of authoritarian leadership on safety compliance behavior and safety participation behavior. Besides, the bias-corrected percentile Bootstrap method test showed (see Table 4) that the mediating effect of trust in leadership between authoritarian leadership and safety compliance behavior is significant (β = –0.044, 95% CI = [–0.077, –0.015]); the mediating effect of trust in leadership between authoritarian leadership and safety participation behavior is also significant (β = –0.043, 95% CI = [–0.076, –0.014]). This analysis thus supports hypotheses 2a and 2b.

**Table 3:** Mediated model test for trust in leadership.

**Variable**	**Safety compliance behavior**			**Safety compliance behavior**			**Safety participation behavior**			**Safety participation behavior**			**Trust in leadership**		
	**β**	**SE**	***t***	**β**	**SE**	***t***	**β**	**SE**	***t***	**β**	**SE**	***t***	**β**	**SE**	***t***
Gender	0.094	0.065	1.455	0.044	0.061	0.709	0.008	0.073	0.110	−0.042	0.070	−0.600	0.147	0.062	2.366^*^
Age	−0.018	0.011	−1.674	−0.020	0.010	−1.972^*^	−0.034	0.012	−2.809^**^	−0.036	0.012	−3.106^**^	0.006	0.010	0.572
Education	0.018	0.046	0.395	0.0188	0.043	0.426	−0.034	0.051	−0.672	−0.034	0.049	−0.694	−0.001	0.044	−0.024
Working years	0.032	0.009	3.704^***^	0.031	0.008	3.849^***^	0.035	0.010	3.663^***^	0.035	0.009	3.762^***^	0.002	0.008	0.208
Authoritarian leadership	−0.192	0.040	−4.816^***^	−0.149	0.038	−3.912^***^	−0.182	0.045	−4.046^***^	−0.139	0.043	−3.199^**^	−0.126	0.038	−3.292^***^
Trust in leadership				0.345	0.039	8.779^***^				0.341	0.045	7.601^***^			
*R* ^2^	0.084			0.185			0.060			0.139			0.034		
F	17.740			8.264			5.714			12.674			3.200		

*N = 636;*

* *p < 0.05;*

** *p < 0.01;*

*** *p < 0.001*.

**Table 4:** Decomposition of indirect, direct and total effects.

**Variable**	**Type of effect**	**Effect value**	**Boot SE**	**Boot LLCI**	**Boot ULCI**	**Relative effect value (%)**
Safety compliance behavior	Indirect effect	−0.044	0.016	−0.077	−0.015	23
	Direct effect	−0.149	0.037	−0.224	−0.079	77
	Total effect	−0.192	0.037	−0.266	−0.120	
Safety participation behavior	Indirect effect	−0.043	0.016	−0.076	−0.014	24
	Direct effect	−0.139	0.043	−0.224	−0.053	76
	Total effect	−0.182	0.044	−0.269	−0.094	

*Boot SE, Boot LLCI, and Boot ULCI refer to the standard error, lower limit, and upper limit of 95% confidence interval of the effect estimated by the percentile Bootstrap method of bias correction, respectively; all values are retained by rounding to two decimals*.

### Moderated Mediating Effect

Based on the above results, the moderating variable locus of control was introduced to construct the final integrated model. Controlling for gender, age, education, and working years, we used authoritarian leadership as the independent variable, locus of control as the moderating variable, trust in leadership as the mediating variable, and safety compliance behavior and safety participation behavior as the dependent variables, and used model 7 in PROCESS to test the moderated mediating effects of the first half of the model path.

The results are shown in Table 5, authoritarian leadership has a significant and negative effect on safety compliance behavior (β = –0.142, *p* < 0.001) and safety participation behavior (β = –0.132, *p* < 0.01), and it also has a significant and negative influence on trust in leadership (β = –0.106, *p* < 0.01). Besides, trust in leadership influences safety compliance behavior (β = 0.343, *p* < 0.001) and safety participation behavior (β = 0.340, *p* < 0.01) significantly and positively, and the interaction term between authoritarian leadership and locus of control influences trust in leadership (β = 0.229, *p* < 0.001) significantly and positively. The locus of control also has a significant and positive effect on trust in leadership (β = 0.123, *p* < 0.05). The above analysis suggests that the locus of control does moderates the first half of the mediating effect.

**Table 5:** Moderated mediating effect.

**Variable**	**Model 1: Safety compliance behavior**			**Model 2: Safety participation behavior**			**Model 3: Trust in leadership**		
	**β**	**SE**	***t***	**β**	**SE**	***t***	**β**	**SE**	***t***
Gender	0.051	0.061	0.837	−0.033	0.070	−0.477	0.171	0.061	2.789^**^
Age	−0.021	0.010	−2.068^*^	−0.037	0.012	−3.187^**^	0.009	0.010	0.883
Education	0.018	0.040	0.445	−0.027	0.046	−0.592	0.020	0.040	0.502
Working years	0.031	0.008	3.903^***^	0.035	0.009	3.858^***^	0.000	0.008	−0.048
Authoritarian leadership	−0.142	0.038	−3.746^***^	−0.132	0.043	−3.062^**^	−0.106	0.040	−2.651^**^
Trust in leadership	0.343	0.039	8.729^***^	0.340	0.045	7.573^***^			
Locus of control							−0.123	0.049	−2.494^*^
Authoritarian leadership × locus of control							0.229	0.056	4.067^***^
R^2^		0.180			0.135			0.062	
F		22.957^***^			16.317^***^			5.922^***^	

*N = 636;*

* *p < 0.05;*

** *p < 0.01;*

*** *p < 0.001*.

To further test whether the moderated mediating effects of this study were valid, we conducted a conditional mediating effects analysis. The results are shown in Table 6, where the mean of locus of control plus or minus one standard deviation represents the “high level of locus of control (i.e., representing internals)” and the “low level of locus of control (i.e., representing externals),” respectively. The results indicated that the indirect effects of authoritarian leadership on safety compliance behavior (β = −0.088, SE = 0.025, 95% CI = [–0.141, –0.044]) and safety participation behavior (β = –0.087, SE = 0.025, 95% CI = [–0.141, –0.417]) through trust in leadership are significant for externals. However, the indirect effects of authoritarian leadership affecting safety compliance behavior (β = 0.015, SE = 0.020, 95% CI = [–0.025, 0.056]) and safety participation behavior (β = 0.015, SE = 0.020, 95% CI = [–0.025, 0.054]) through trust in the leadership are not significant for internals. Besides, the difference between the indirect effects of the high and low groups was equally significant (β = 0.103, SE = 0.030, 95% CI = [0.048, 0.166]; β = 0.102, SE = 0.031, 95% CI = [0.046, 0.165]). This analysis thus verifies hypothesis 3.

**Table 6:** Mediating effects at different levels of locus of control.

**Locus of control**	**Safety compliance behavior**				**Safety participation behavior**		
	**Indirect effect**	**Boot SE**	**Boot LLCI**	**Boot ULCI**	**Indirect effect**	**Boot SE**	**Boot LLCI**	**Boot ULCI**
Eff1 (M-1SD)	−0.088	0.025	−0.141	−0.044	−0.087	0.025	−0.141	−0.042
Eff2 (M)	−0.036	0.017	−0.073	−0.006	−0.036	0.017	−0.073	−0.006
Eff3 (M+1SD)	0.015	0.020	−0.025	0.056	0.015	0.020	−0.025	0.054
Eff2-Eff1	0.052	0.015	0.024	0.083	0.051	0.015	0.023	0.083
Eff3-Eff1	0.103	0.030	0.048	0.166	0.102	0.031	0.046	0.165
Eff3-Eff2	0.052	0.015	0.024	0.083	0.051	0.015	0.023	0.083

*Boot SE, Boot LLCI, and Boot ULCI refer to the standard error, lower limit, and upper limit of 95% confidence interval of the effect estimated by the percentile Bootstrap method of bias correction, respectively; all values are retained by rounding to two decimals*.

**Figure 2:**
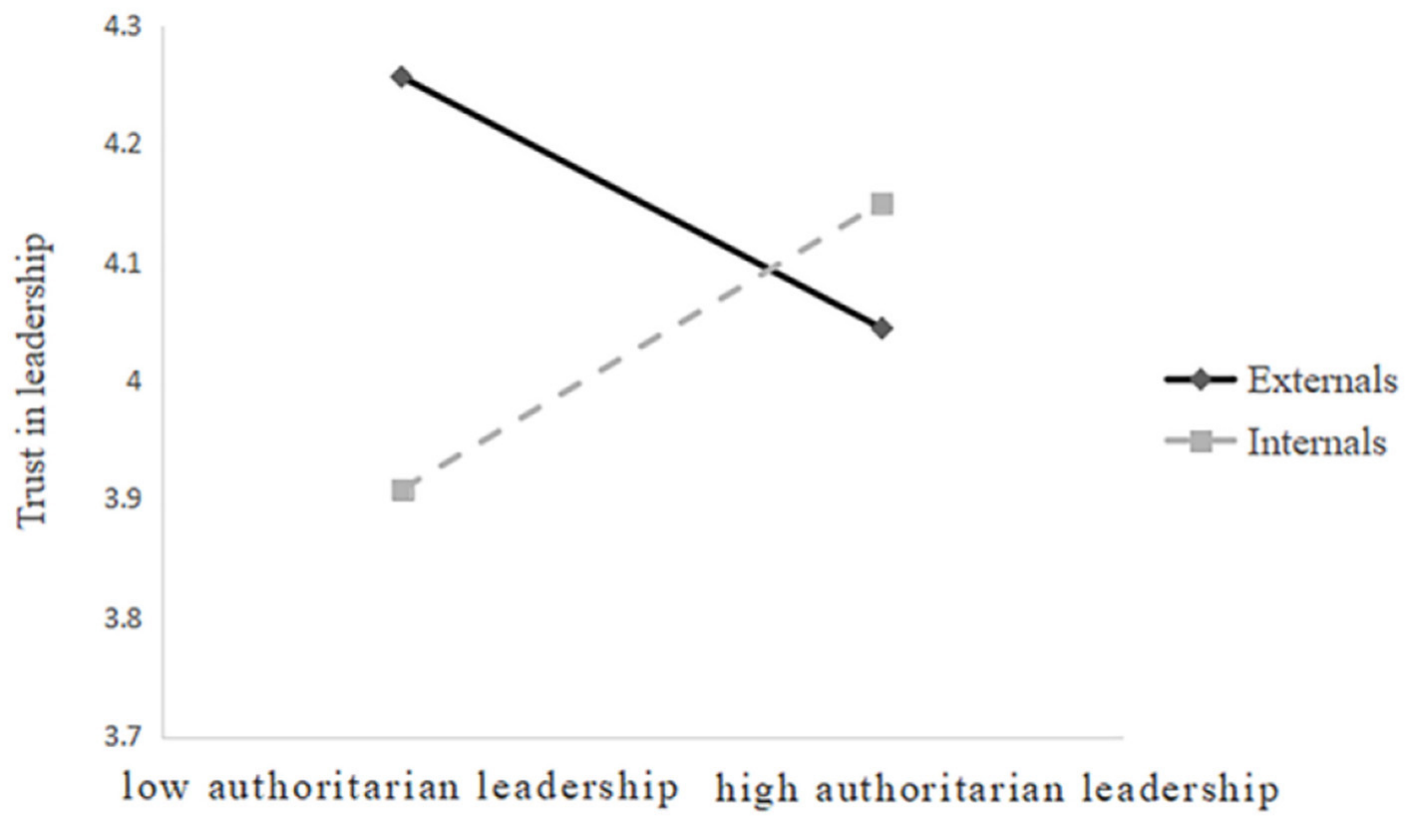
Moderating effects of locus of control on the relationship between authoritarian leadership and trust in leadership.

To further describe the specific pattern of the moderating effect of locus of control, we also conducted a simple effects analysis. As shown in Figure 2, the results of the simple slope test indicated that for externals, the negative predictive effect of authoritarian leadership on trust in leadership is significant, and they are less likely to build trust with their leaders under authoritarian leadership; for internals, the positive predictive effect of authoritarian leadership on trust in leadership is significant, and they are more likely to build trust with their leaders in the same situation.

## Discussion

Chairman Xi Jinping pointed out in the report of the 19th National Congress of the Communist Party of China that it is necessary to establish the concept of safe development, promote the idea of life first and safety first, improve the public safety system, refine the responsibility system for safe production, and resolutely curb serious safety accidents. Production safety management is an important part of the public safety system, which aims to fully protect the occupational health of employees and avoid the threat to their lives and health and socio-economic loss of enterprises due to work-related injuries, occupational diseases and other potential hazardous factors. In the case of high-risk enterprises such as oil, mining, and chemical industries, we should focus on the safety behavior of employees because of their special, complex and dangerous working environment. This article explores the influence and mechanism of authoritative leadership style on the safety behavior of petroleum employees at the enterprise and individual levels, with the aim of further expanding theoretical research in the field of employee safety behavior and, more importantly, providing relevant policy recommendations for the occupational health of employees in high-risk enterprises and the safety management of such enterprises.

First, this study has empirically examined that authoritarian leadership is significantly and negatively related to employees' safety compliance behavior and safety participation behavior, which supports hypothesis 1a and 1b.We explain this result in terms of the social exchange theory proposed by Homans (48). According to social exchange theory, people will analyze the costs and benefits of a certain relationship in the process of social interaction, and a satisfactory social relationship should ensure the balance between “costs” and “benefits.” However, authoritarian leaders, tend to exercise strict domination and control over their employees and rarely interact with them in a positive way. They do not listen to employees' demands, do not pay attention to employees' expectations and needs, and even ignore their personal development (45), which makes it difficult to balance the social exchange between employees and leaders. Faced with low “benefits” from authoritarian leaders, employees will reduce their work engagement (57, 58), increase their counterproductive work behavior, and thus decrease their attention to safety norms, showing negligence and disregard for safety compliance regulations etc. behavior. That is, authoritarian leadership will eventually have a negative impact on employees' safety compliance behavior. In addition, employees will also cut down on their voice behavior and organizational citizenship behavior (64, 126), which is manifested in the field of safety production, i.e., employees will not pose meaningful opinions on the improvement of corporate safety production or take the initiative to supervise and help colleagues to operate production machines safely. In other words, authoritarian leadership will also eventually have a negative impact on employees' safety participation behavior. Previous studies have mainly focused on the role of transformational leadership, etc. in employees' safety behavior based on the Western cultural context (17). While the present study focuses on the relationship between one of the dimensions of paternalistic leadership- authoritarian leadership formed based on traditional Chinese cultural values and employee safety behavior. In addition, this study further explores the role of authoritarian leadership on both two dimensions of safety behavior. This study fills a gap in relevant field and also expands the relevant elaboration of social exchange theory in safety production, providing support for its application in future research and practical contexts. As for the policy recommendations, we believe that as far as individuals are concerned, leaders should be aware of their behaviors and actions in the work and change their authoritarian leadership style. As far as companies are concerned, corporate training for leaders can effectively improve their leadership effectiveness (127). Therefore, companies should pay attention to developing leaders' empowerment and service mindset and encourage them to create a highly empowered and service-oriented organizational climate and organizational culture in the organization. Appropriate empowerment by leaders can increase employees' autonomy and intrinsic motivation to work, thereby facilitating their safety participation behavior (128). In addition, the leaders' emphasis on emotional communication with employees and caring for their needs will also help to increase employees' job satisfaction and engagement, so that they can better participate in safety compliance behavior. Finally, as far as society is concerned, the state should deepen the concept of “service-oriented government” transformation, so as to positively influence the change of leadership in high-risk enterprises and promote the transformation of high-risk enterprise leaders to a “service, efficient, and safety-oriented” leadership style. In addition, social departments such as the Health and Welfare Commission and the Ministry of Human Resources and Social Security should strengthen the publicity and construction of production safety culture and production safety atmosphere in order to improve the intrinsic safety awareness of enterprises and employees from soft culture and reduce the occurrence of safety accidents.

Second, this study has examined the mediating role of trust in leadership between authoritarian leadership and safety compliance behavior as well as safety participation behavior. In other words, this study concluded that authoritarian leadership leads to a decrease in trust in leadership, thereby having a negative impact on safety compliance behavior and safety participation behavior, which supports hypothesis 2a and 2b. Based on the idea that leader traits influence the process of forming trust in leadership, authoritarian leaders traits such as harshness, high power distance, and impersonality would reduce employees' cognitive trust in leaders (74); In addition, based on the idea that leader-employee relationship influence the process of forming trust in leadership, authoritarian leaders' autocratic behavior and unwillingness to empower employees etc. can seriously hinder the establishment of high-quality relationship between leaders and employees, thereby impeding the affective trust between leaders and employees (27). That is, authoritarian leadership would reduce employees' trust in leadership. Low level of trust between authoritarian leaders and employees is not beneficial to the establishment of high-quality social exchange relationship between them, in which case employees will decrease their work engagement (74) and the degree of effort and seriousness to the tasks assigned by the leader (85); In addition, employees' supportive behavior and proactive service to the organization can be also negatively affected, all of which can reduce their own safety compliance behavior and safety participation behavior. This result not only reveals the internal mechanism between authoritarian leadership and safety behavior, but also verifies that trust in leadership affects employees' behavior in the workplace, which provides new ideas for future research that more attention could be paid to the relationship between employees and leaders. As for the policy recommendations, we believe that as far as individuals are concerned, leaders are expected to improve their leadership skills in the management by demonstrating to their subordinates traits such as fairness, reliability, kindness, and competence. Leaders should also actively communicate with employees, care about their needs, and provide them with material help and emotional support as much as possible, so as to enhance the cognitive trust and affective trust with employees (27, 74). According to the social learning theory proposed by Bandura (129), leaders leading by example in safety is the key to increasing the level of trust employees have in their leaders. Through field research we learned that in oil companies, if a safety accident occurs, leaders will promptly lead their staff to conduct an analysis of the cause of the accident, with the aim of making the staff learn from it and reducing and avoiding such accidents in their future work. Therefore, as far as companies are concerned, they should establish similar work rules and regulations such as safety accident responsibility system, safety accident analysis workflow, safety accident warning and prevention propaganda, which can strengthen the collaboration and cooperation between leaders and employees and improve mutual trust with the ultimate goal of reducing safety accidents.

Finally, this study also examined the moderating role of locus of control between authoritarian leadership and trust in leadership. That is, the work style of authoritarian leadership is more likely to reduce employees' trust in leadership when they are externals, which supports hypothesis 3. Looking back on previous studies, researchers have found that individual differences in locus of control influence their responses to authoritarian leadership (40). We explored this further and found that it has a moderating effect between authoritarian leadership and employees' trust in leadership. According to the locus of control theory (130), internals and externals have different understandings of locus of control, and thus their attitudes and behaviors toward things are varied. Internals tend to blame themselves for their own factors, such as their poor performance, that cause leaders to exert authoritarian behavior on them, and to create a sense of shame that the image of dedicated employees is damaged under the reprimand of authoritarian leaders, all of which force internals to be more proactive in their work and earn the trust of their leaders (103, 105, 112). In contrast, externals, due to self-serving bias, blame the leader's authoritarian behavior on their own personal factors, and therefore do not experience the sense of self-blame and shame experienced by the internals. Besides, externals tend to choose passive avoidance in this low-quality relationship with the authoritarian leader rather than proactively demonstrating better work behavior and enhancing trust with the authoritarian leader (99, 104, 106, 113). Our study expands the application of the locus of control theory in safety production. Moreover, we further clarifies the mechanism of authoritarian leadership on safety behavior, i.e., the locus of control may be an important moderating factor that moderates the path of authoritarian leadership on employees' trust in leadership. As for the policy recommendations, we believe that as far as companies are concerned, they should focus on screening people who possess the traits of high LOC during the written test and interview process of recruitment and selection. In addition, enterprises should also attach importance to the training of internals, such as: appropriate authorization, carry out some group activities to improve the self-efficacy of employees, improve the non-material incentive mechanism, arrange training courses on the locus of control to employees, and put more emphasis on human intrinsic factors in the analysis of safety accidents.

## Limitations and Future Research

First, the data in this paper comes from a state-owned petroleum enterprise in China. Since the state attaches great importance to the safety production of petroleum enterprises, this enterprise has a very strict grip on safety production, so the safety awareness of employees in this enterprise may be higher than that of employees in other enterprises or industries. Therefore, future studies should extend the sample to other enterprises or industries to expand the scope of application of the findings.

Second, in order to avoid common method bias, the study divided the data collection process into two time points for collection, but still the results of employee self-assessment. Although we have emphasized the authenticity and anonymity of the questionnaire responses, the influence of biases such as social approvability on the results cannot be excluded. Therefore, future studies can collect data from multiple perspectives, such as adding leaders' evaluations or colleagues' mutual evaluations. Besides, the current study is essentially just a cross sectional study, meaning that causality cannot be inferred. Future researchers can use longitudinal research such as cross-lagged method to test for more precise causality among all variables.

Third, authoritarian leadership in the Chinese context may differ from authoritarian leadership in other countries with its unique traditional Chinese cultural characteristics. Therefore, future research should take cross-cultural factors into account and examine safety behavior across countries or across ethnic groups.

## Conclusion

- Authoritarian leadership is significantly and negatively correlated with safety compliance behavior and safety participation behavior;
- Employees' trust in leadership mediates the relationship between authoritarian leadership and safety compliance behavior, as well as safety participation behavior;
- Locus of control moderates the first half of the pathway through which authoritarian leadership affects employees' safety behavior through trust in leadership. For externals, authoritarian leadership has a stronger negative impact on their trust in leadership, which in turn can reduce their safety compliance behavior and safety participation behavior.

## Data Availability Statement

The original contributions presented in the study are included in the article/supplementary material, further inquiries can be directed to the corresponding author/s.

## Ethics Statement

The studies involving human participants were reviewed and approved by the Ethics Committee of Shandong Normal University. The patients/participants provided their written informed consent to participate in this study.

## Author Contributions

DW: model building, writing, revisions, data collection and analysis. LW: writing, revisions and data analysis. SW: writing, data analysis. PY: model building. HS: writing, data collection and analysis. XJ: data collection. YH: model building, revisions, supervision. All authors contributed to the article and approved the submitted version.

## Funding

This study was supported by General Project of Education of the National Social Science Fund: The development, influencing factors, and intervention system of middle school students' moral shading: Based on decision-making process (Grant No. BEA210108).

## Conflict of Interest

XJ was employed by Shengli Petroleum Engineering Yellow River Drilling Corporation. The remaining authors declare that the research was conducted in the absence of any commercial or financial relationships that could be construed as a potential conflict of interest.

## Publisher's Note

All claims expressed in this article are solely those of the authors and do not necessarily represent those of their affiliated organizations, or those of the publisher, the editors and the reviewers. Any product that may be evaluated in this article, or claim that may be made by its manufacturer, is not guaranteed or endorsed by the publisher.
